# Myoepithelioma within the carpal tunnel: a case report and review of the literature

**DOI:** 10.1186/1477-7800-6-15

**Published:** 2009-09-09

**Authors:** Jonathan CM Clark, Stuart J Galloway, Stephen M Schlicht, Ross PV McKellar, Peter FM Choong

**Affiliations:** 1 Department of Orthopaedics and Department of Surgery, University of Melbourne, St Vincent's Hospital, Melbourne, Australia; 2 Department of Anatomical Pathology, St Vincent's Hospital, Melbourne, Australia; 3 Department of Radiology, St Vincent's Hospital, Melbourne, Australia; 4 Bone and Soft Tissue Sarcoma Service, Peter MacCallum Cancer Centre, Melbourne, Australia

## Abstract

Myoepitheliomas of the extremity are rare and usually benign, while a minority display malignant features. This case demonstrates the diagnosis and management of myoepithelioma within the carpal tunnel. Clinical and radiological tumour features were evaluated. Hematoxylin and eosin stained tumour sections were examined, and immunohistochemistry was performed. Histology revealed a nodular mass of epithelioid cells in clusters within a myxoid/chondroid stroma. No mitoses were noted. Cytokeratins, neuron-specific enolase, synaptophysin, glial fibrillary acidic protein, and S100 were positive on immunohistochemistry. A literature review revealed very few prior reports of myoepithelioma in the wrist, and limited data concerning any relationship between recurrence and quality of surgical margins. In this case, wide local excision would have significantly compromised dominant hand function, and therefore a marginal excision was deemed appropriate in the context of bland histological features. Surgical margins noted in future case reports will aid clinical decision making.

## Introduction

Myoepithelioma, also known as parachordoma, is a rare soft-tissue tumour more commonly associated with the salivary gland [1]. It is also found in the extremities. Only three cases have been reported in the wrist (Table 1), and only one of these caused median nerve compression [2]. Myoepithelioma may be found attached to tendon sheaths and shares similar radiological findings to ganglions, pigmented villonodular synovitis, and extraskeletal myxoid chondrosarcoma (ESMC). Histological diagnosis of myoepithelioma can be difficult and the histological criteria for malignancy are still being defined. These difficulties of diagnosis and the rarity of the tumour provide a management challenge. This case report and literature review outlines the process of diagnosing myoepithelioma in the carpal tunnel, specifically focusing on differential diagnosis and management principles.

**Table 1:** A summary of reported myoepithelioma/parachordoma cases in the upper limb (excluding the shoulder girdle)

**Authors**	**Site**	**Length of History**	**Histology**	**Surgery** **(margins)**	**Local Recurrence**	**Metastasis**
Clabeaux et al 2008 [23]	Distal triceps	3 m	Parachordoma, (nuclear atypia, one mitosis per 20 HPF)	Wide	None (3 m post op)	None (3 m post op)
Pilavaki et al 2007 [24]	Hand (hypothenar, subcutaneous)	4 y	Myoepithelioma (no mitoses)	Excisional biopsy (marginal margins)	None (9 m post op)	None (9 m post op)
Gleason and Fletcher 2007 [5]	Hand	NA	Myoepithelial carcinoma	NA	Yes (7 y post op)	No
	Wrist	NA	Myoepithelial carcinoma	NA	NA	NA
Hamada et al 2006 [25]	Hand (thenar eminence)	3 y	Myoepithelioma	NA	NA	None (at diagnosis)
Harada et al 2005 [8]	Forearm	14 y	Myoepithelioma (nuclear atypia, 2-3 mitoses per 10 HPF)	Local excisional biopsy	NA	Lung mets (6 m post op)
Colombat et al 2003 [2]	Wrist	10 y	Myoepithelioma	Clear margins	None (5 m post op)	None (5 m post op)
Šeparović et al 2001 [26]	Hand	2 y	Parachordoma	Excisional biopsy (unknown margins)	None (20 m post op)	None (20 m post op)
Folpe et al 1999 [27]	3 cases in the arm (deep to superficial fascia)	NA	Parachordoma (< 1 mitosis per 20 HPF)	NA	None (min 4 m follow up)	None (min 4 m follow up)
Michal et al 1999 [28]	Proximal thumb	NA	Myoepithelioma	NA	None (19 y post op)	None (19 y post op)
Imlay et al 1998 [29]	Forearm (subcutaneous)	NA	Parachodroma No mitoses	NA	None (12 m post op)	None (12 m post op)
Kilpatrick et al 1997 [30]	Hand	NA	Myoepithelioma (ductal differentiation, < 2 mitoses per 10 HPF)	NA	Yes (14 m post op)	NA
	Finger	3 m	Myoepithelioma (myxoid features, < 2 mitoses per 10 HPF)	NA	None (2 y post op)	None (2 y post op)
Fisher and Miettinen 1997 [31]	Triceps	6 m	Parachordoma (rare mitoses, mild nuclear pleomorphism)	NA	None (2 y post op)	None (2 y post op)
	Wrist (attached to tendon of flexor carpi ulnaris)	NA	Parachordoma (rare mitoses, mild nuclear pleomorphism)	NA	NA	NA
Nietzabitowski et al 1995 [9]	Palm	4 y	Parachordoma	NA	Yes (3 m post op)	None (23 post op)
Sangueza and White 1994 [32]	Finger (adjacent to proximal IP joint)	5 y	Parachordoma (slight nuclear pleomorphism, no mitoses)	NA	NA	NA
Dabska 1977 [33]	Arm	7 m	Parachordoma	NA	None (10 y post op)	None (10 y post op)
	Distal radius	2 m	Parachordoma	Likely wide margins	None (6 y post op)	None (6 y post op)
	Finger	NA	Parachordoma	Likely marginal or intralesional	None (5 y post op)	None (5 y post op)

NA (not available), y (years), m (months), HPF (high power fields)

In three cases there was local recurrence and one case resulted in lung metastases.

### Case presentation

A 36 year old Caucasian male presented with a mass in the right wrist and paraesthesia in the distribution of the median nerve. The mass had been noticed four years prior to presentation and over this time had increased in size. On examination the mass projected from the volar aspect of the wrist but was poorly demarcated. It was firm to palpate and non-tender. Hand examination revealed attachment of the mass to the flexor digitorum profundus tendon of the index finger. Paraesthesia was exacerbated by repetitive use of all the long flexor tendons. No thenar eminence wasting was noted but some mild clinical weakness was evident in this muscle group.

**Figure 1:**
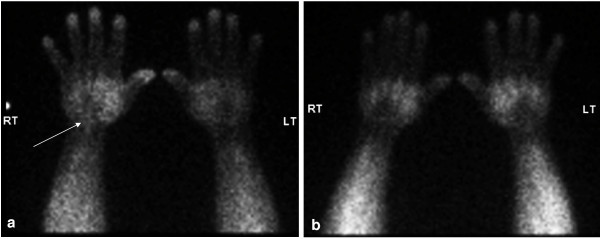
**Thallium-201 scintigraphy at a) 30 minutes demonstrates focal uptake in the right wrist (arrow), and b) at four hours, wash-out in this area had occurred**.

An initial MRI scan performed outside our institution demonstrated a T2 hyperintense lesion, 50 mm long by 14 mm wide, arising from the index finger profundus tendon within the palm and displacing the median nerve. Thallium-201 scintigraphy (Fig. 1) demonstrated initial tracer uptake in a small focus corresponding with the mass described on MRI. Images at four hours post infusion revealed complete washout of the abnormal tracer activity in this region. These images indicated a likely benign diagnosis of a ganglion or pigmented villonodular synovitis (PVNS), while synovial sarcoma was considered to be unlikely given the thallium washout at four hours.

A CT guided core-biopsy of the lesion was performed. The lesion could not be aspirated and two 18 gauge cores of gelatinous material were taken. Microscopic examination of these cores revealed a chondroid matrix and bland cells suggestive of synovial chondromatosis.

**Figure 2:**
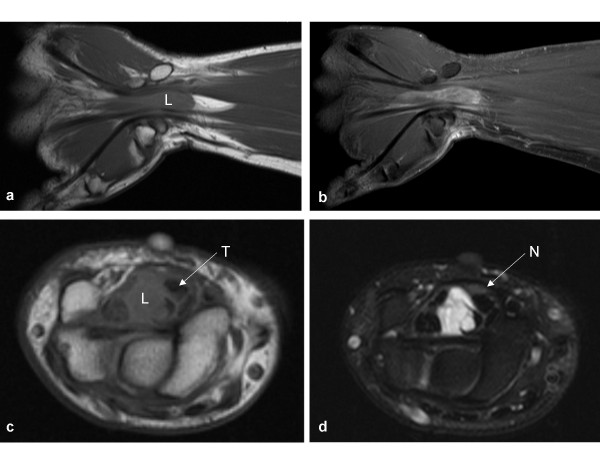
**MRI of the lesion (L) shows it a) attached to the index finger profundus tendon in the coronal plane on T1 weighted imaging, b) demonstrating septal enhancement post contrast administration, c) displacing the superficialis tendons (T) to the middle and index fingers radially on axial T1 imaging, and d) showing heterogeneous T2 hyperintensity again in the axial plane, with the compressed median nerve (N) more clearly visualised**.

A second MRI scan (Fig. 2), performed 12 months later, showed the tumour size had not changed from the previous scan and the long axis of the mass remained parallel to that of the profundus tendon. Fluid signal was noted predominantly but with the addition of irregular T2 hypointense septations within it, which enhanced with contrast. The mass was also found to displace flexor digitorum superficialis (FDS) tendons to the ring and little fingers in a volo-ulnar direction and the FDS tendons to the index and middle finger in a volo-radial direction (Fig. 2c, d). The median nerve was compressed anteriorly between the FDS tendon slips to the middle and index fingers and the overlying flexor retinaculum (Fig. 2c, d). Associated T2 hyperintensity of the median nerve was demonstrated over its passage through the carpal tunnel.

An excisional biopsy with marginal margins was subsequently performed. A longitudinal incision was made over the volar aspect of the wrist and hand, entering the carpal tunnel from its radial aspect. The mass was confirmed to be attached to the profundus tendon sheath of the index finger and was excised from this surface.

## Materials and methods

Hematoxylin and eosin-stained sections were examined. Immunohistochemistry, involving streptavidin-biotin peroxidase, was performed on formalin-fixed, paraffin-embedded tumour specimens to keratins AE1/AE3 (Novo Castra, Newcastle Upon Tyne, UK; 1/80 v), neuron-specific enolase (NSE) (DakoCytomation, Denmark; 1/1000 v), glial fibrillary acid protein (GFAP) (DakoCytomation, Denmark; 1/5000 v), S100 protein (DakoCytomation, Denmark; 1/8000 v), smooth muscle actin (SMA) (DakoCytomation, Denmark; 1/200 v), and synaptophysin (DakoCytomation, Denmark; 1/100 v). Diaminobenzidine was used as the chromogen.

### Pathological findings

**Figure 3:**
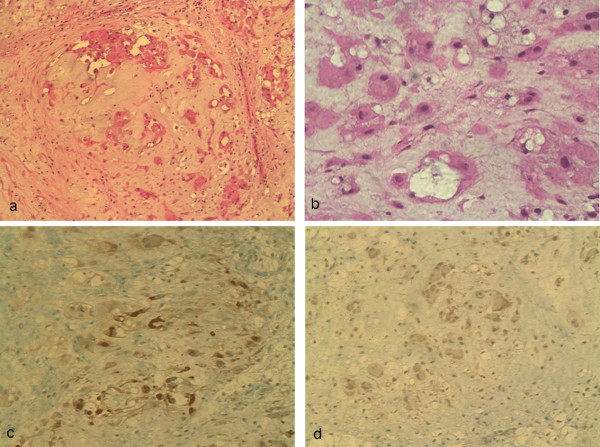
**Histopathology of the excisional biopsy specimen demonstrates a) lobules of chondroid/myxoid matrix with embedded vacuolated cells and plasmacytoid cells (100×), b) these same cell populations are shown at high-power (400×), c) positive cytokeratin AE1-3 staining (200×), and d) weak GFAP staining (200×)**.

Histological examination revealed a nodular lesion composed of large epithelioid cells, singly and in clusters floating in a loose myxoid/chondroid stroma (Fig. 3a, b). The cells contained abundant eosinophilic cytoplasm, some of which were multivacuolated or "physaliferous". The nuclei showed mild atypia but no mitoses were seen.

Peripheral tumours with chondroid stroma include chondrosarcoma, ESMC, and ossifying fibromyxoid tumour (OFT). Chondrosarcoma was excluded by positive keratin markers and OFT was excluded by the clinical appearance, soft consistency and lack of ossifying and firm fibrous areas. ESMC demonstrates similar clinical and histological features to the reported lesion, but myoepithelioma was favoured, because of positive cytokeratins, GFAP staining (Fig. 3c, d), and S100 [3,4]. Table 2 summarises the immunohistochemical profile of our reported tumour, and the percentage of previously reported myoepitheliomas and ESMC staining positive for the same markers.

**Table 2:** A profile of immunohistochemical markers staining positive or negative in the reported tumour tissue

**Antibody**	**Reported tumour**	**Expected ME **[4]	**Expected ESMC **[3]
Cytokeratin	+	77%	1%
NSE	+	+ [1]	54%
SYN	+/-	+ [1]	59%
GFAP	+	46%	2%
SMA	-	36%	6%
S100	+	87%	31%

ME (myoepithelioma), ESMC (extra-skeletal myxoid chondrosarcoma)

This is compared with the percentage of positive staining reported in other cases of myoepithelioma and extraskeletal myxoid chondrosarcoma.

## Discussion

Myoepithelial tumours are a class of primary soft tissue tumours that are becoming more frequently recognised, probably as a result of the increasing number and range of immunoperoxidase stains. The World Health Organisation (WHO) classification of tumours gives mixed tumour and parachordoma as synonyms of myoepithelial tumours [1]. Approximately 40% of tumours within this spectrum are considered malignant [4], and have been referred to by Hornick and Fletcher as myoepithelial carcinomas. This malignant variant is more likely to occur in children [5]. The term myoepithelioma is more synonymous with a benign, although potentially locally recurrent, lesion.

Myoepitheliomas are found in the deep tissue planes in the extremities. Tumours in cutaneous planes are considered tumours of skin appendages rather than primary soft tissue tumours. A rare case, reported by Alberghini and co-workers [6], was found to arise from bone and metastasised to the lung.

In the myoepithelioma series of Hornick and Fletcher [4] the gender distribution was essentially equal with 52% male and 48% female. The age range was 3 to 83 years, with the mean at 38 years and most frequently presenting between the 3^rd ^and 5^th ^decades.

The defining histological characteristics of these tumours are a lobular pattern on low power microscopy, a myxoid or chondroid matrix and an epithelial component which may have plasmacytoid, spindle and clear cell features (Fig. 3). Immunoperoxidase staining is essential to the diagnosis and the characteristic features are shown in Table 2.

There are a number of important differential diagnoses for myoepithelioma arising in the carpal tunnel. Extraskeletal myxoid chondrosarcoma (ESMC), which has already been mentioned, is often associated with tendons supplying the hand, and can show very similar myxoid and chondroid matrix features to myoepithelioma. In our patient it was excluded mainly by positive cytokeratin markers [3]. We were particularly concerned about ruling out malignant chondroid lesions given that an initial biopsy had shown synovial chondromatosis, and this can be a precursor for chondrosarcoma [7]. Limited evidence suggests that transformation of benign lesions within the myoepithelioma spectrum is possible [4,8,9]. In hindsight it is clear that the initial core biopsy was not representative and showed tissue at the junction of tumour and normal tissue. Therefore, it is most likely that such inherent difficulties in tumour biopsy contributed to a revised diagnosis rather than any transformation within the lesion.

Synovial sarcoma arises in similar peripheral locations to myoepithelioma and can also demonstrate myxoid areas and positive cytokeratin markers. It is differentiated from myoepithelioma on the basis of negative SMA and GFAP staining [10], and also its predominantly fibroblastic/spindle cell cytology, although there are biphasic features in 15% [11].

Malignant peripheral nerve sheath tumour (MPNST) is also an important diagnosis to exclude given the proximity to the median nerve, and its usual epithelioid morphology. MPNST also shares S100 expression and a myxoid stroma [12] with myoepithelioma. A key diagnostic feature of MPNST is the observation of tumour arising directly from a peripheral nerve, or a benign neural tumour on imaging. While our reported tumour was impinging on the median nerve, it appeared to arise from tendon on both MRI and intraoperatively.

Epithelioid sarcoma, again a key differential for a mass in the wrist or hand, while cytokeratin positive [11], is easier to differentiate from myoepithelioma because it generally lacks myxoid and chondroid stroma.

Ossifying fibromyxoid tumour (OFT) is a benign, although locally recurring lesion, with distinct similarities to myoepithelioma such as a lobular architecture, epithelioid component, myxoid matrix and focal bone formation [13]. Furthermore, OFT is S100 positive, and occasionally positive for cytokeratins and GFAP. The lesion presents with a firm, fibrous capsule and an ossifying rim in around half of cases [13], and while myoepithelioma may also contain bone, it is only present in around 10% of cases [4]. Another distinguishing feature, although not performed in our case, is absent p63 staining [13], in contrast to 23% of myoepitheliomas being p63 positive [4].

In regard to clinicopathological correlation, Hornick and Fletcher [4] studied 101 cases of myoepithelioma with their main criteria for a malignant classification being moderate to severe cytological atypia. These tumours were noted to recur locally in 42%, and metastasise in 32% of cases. On the other hand, cytologically bland tumours only recurred locally in 18% and none metastasised. In myoepithelioma of the upper limb, the risk of local recurrence appears to be even less with only 3 of 20 reported cases (15%) recurring locally (Table 1). Surprisingly, infiltrative features on histology (noted by Savera et al [14]) do not correlate with recurrence and metastasis, and very little data exists on the influence of surgical margins (Table 1).

Mitotic rate in malignant salivary myoepitheliomas has been thought to be associated with an increased risk of death from disease although this is only based on a very small number of cases [15]. Darvishian and Lin [16] described malignant features of myoepithelial carcinoma arising mainly in the salivary glands, being pleomorphism, prominent nucleoli, mitotic figures, coarse chromatin and necrosis. In contrast, eight related benign lesions (all pleomorphic adenoma) demonstrated no evidence of these features. While myoepitheliomas and mixed tumours are well recognised in salivary glands, given the relatively small published numbers of soft tissue myoepithelial tumours, it is not yet established that prognostic features for salivary tumours are directly applicable to these peripheral soft tissue tumours.

Space-occupying lesions are an uncommon cause of carpal tunnel syndrome, accounting for around 5% of cases, and strongly linked to a unilateral presentation with 35% of these patients having a space-occupying lesion [17]. This emphasises the importance of adequate investigation in unilateral carpal tunnel syndrome. Tumours reported in the carpal tunnel include ganglions [17], lipoma [18], haemangioma [19], fibroma [20], and synovial sarcoma [21]. Incidentally, extraskeletal chondroma, which is related to our initial diagnosis of synovial chondromatosis, has also been published in regard to a carpal tunnel location and association with profundus tendons [22]. The only other published case of myoepithelioma in the wrist causing median nerve compression [2] was similar to this reported case in having mild nuclear atypia, few mitoses, and being S100 and cytokeratin positive but SMA negative. A local but complete excision demonstrated no evidence of recurrence at 5 months post surgery.

In the carpal tunnel, the major dilemma associated with the resection of lesions with variable malignant potential concerns the option of curative surgery with wide margins which will permanently compromise hand function, versus a marginal/intralesional excision, which requires accepting a degree of recurrence risk and the potential for re-excision. As discussed, myoepithelial tumours can display subtle features of malignancy, and there is a reported 18% risk of local recurrence in lesions with low-grade cytology [4]. Hornick and Fletcher demonstrated no correlation between quality of margins and recurrence risk [4], and so a clear consensus on surgical management is lacking, although wide margin resection appears to be well utilised [23]. In our case, the decision to perform a marginal excision was much easier as it was based on the perception that we were operating on synovial chondromatosis. In retrospect, and on the basis of Hornick and Fletcher's data, we still see this as the best option for histologically bland myoepithelioma within the carpal tunnel.

## Conclusion

This case demonstrates the challenge of diagnosing myoepithelioma, and differentiating it from other benign and malignant tumours. It shows that even when using CT guided core biopsy, a widely respected diagnostic technique, tissue samples may not adequately represent the true diagnosis. The difficulty in diagnosing myoepithelioma is also related to its rarity and similarity to ESMC. Differentiation relies on meticulous scrutiny of cell morphology and immunohistochemistry. While distal upper limb myoepithelioma is likely to be low grade or benign, there are still relatively few cases reported in this location, and so careful follow-up is advised.

## Competing interests

The authors declare that they have no competing interests.

## Authors' contributions

All listed authors have made a significant intellectual contribution to the preparation and drafting of this manuscript.

## Consent

Written informed consent was obtained from the patient for publication of this case report and the accompanying images.
